# Supplementary material for: Functional interdependence of the actin nucleator Cobl and Cobl-like in dendritic arbor development
Source: eLife. 2021 Jul 15;10:e67718. doi: 10.7554/eLife.67718 (PMC8282341; doi:10.7554/eLife.67718)
Supplement: Transparent reporting form [file elife-67718-transrepform.docx]

***eLife’s* transparent reporting form**

We encourage authors to provide detailed information *within their submission* to facilitate the interpretation and replication of experiments. Authors can upload supporting documentation to indicate the use of appropriate reporting guidelines for health-related research (see [EQUATOR Network](http://www.equator-network.org/%20)), life science research (see the [BioSharing Information Resource](https://biosharing.org/)), or the [ARRIVE guidelines](http://www.plosbiology.org/article/info:doi/10.1371/journal.pbio.1000412) for reporting work involving animal research. Where applicable, authors should refer to any relevant reporting standards documents in this form.

If you have any questions, please consult our Journal Policies and/or contact us: [editorial@elifesciences.org](mailto:editorial@elifesciences.org).

**Sample-size estimation**

- You should state whether an appropriate sample size was computed when the study was being designed
- You should state the statistical method of sample size computation and any required assumptions
- If no explicit power analysis was used, you should describe how you decided what sample (replicate) size (number) to use

Please outline where this information can be found within the submission (e.g., sections or figure legends), or explain why this information doesn’t apply to your submission:

This information can be found in the *Material and Methods* section (paragraph *Statistical analyses and sample-sited estimation*). It reads:

*No explicit power analyses were used to compute and predefine required sample sizes. Instead, all neuronal analyses were conducted by systematic sampling of transfected cells across coverslips to avoid any bias. Morphometric analyses were then conducted by using IMARIS software.*

*All data were obtained from 2-5 independent neuronal preparations seeded onto several independent coverslips for each condition for transfection and immunostaining. For each condition, n numbers of individual neurons ranging from about 30 to 40 were aimed for to fully cover the biological variances of the cells. Higher n numbers yielded from the systematic sampling were accepted, too (e.g. see the control in Figure 1G-I (n=45) and in Figure 1N-P (65)). Lower n numbers were only accepted for the established Cobl overexpression phenotype (n=24) and the Cobl-like-mediated suppression if it (also n=24), as results were clear and sacrificing further rats for further primary neuron preparations could thus be avoided (Figure 1N-P).*

*All n numbers of independent biological samples (i.e. neurons) or biochemical assays are given directly in the figures.*

**Replicates**

- You should report how often each experiment was performed
- You should include a definition of biological versus technical replication
- The data obtained should be provided and sufficient information should be provided to indicate the number of independent biological and/or technical replicates
- If you encountered any outliers, you should describe how these were handled
- Criteria for exclusion/inclusion of data should be clearly stated
- High-throughput sequence data should be uploaded before submission, with a private link for reviewers provided (these are available from both GEO and ArrayExpress)

Please outline where this information can be found within the submission (e.g., sections or figure legends), or explain why this information doesn’t apply to your submission:

All n numbers are reported directly in the figures of the manuscript.

As stated in the *Material and Methods* section (paragraph *Statistical analyses and sample-sited estimation*) of the manuscript, all n numbers reported represent numbers of independent biological samples (i.e. neurons) or biochemical assays, as additional replicates to minimize measurement errors were not required because the technical errors were small in relation to the biological/biochemical variances.

The nummerical data of the wealth of quantitative evaluations included in the manuscript is provided as Supplementary Source data (Excel format).

As also stated in the *Material and Methods* section (paragraph *Statistical analyses and sample-sited estimation*) of the manuscript, outliers or strongly scattering data reflect biological variance and were thus not excluded from the analyses.

**Statistical reporting**

- Statistical analysis methods should be described and justified
- Raw data should be presented in figures whenever informative to do so (typically when N per group is less than 10)
- For each experiment, you should identify the statistical tests used, exact values of N, definitions of center, methods of multiple test correction, and dispersion and precision measures (e.g., mean, median, SD, SEM, confidence intervals; and, for the major substantive results, a measure of effect size (e.g., Pearson's r, Cohen's d)
- Report exact p-values wherever possible alongside the summary statistics and 95% confidence intervals. These should be reported for all key questions and not only when the p-value is less than 0.05.

Please outline where this information can be found within the submission (e.g., sections or figure legends), or explain why this information doesn’t apply to your submission:

The statistical tests used are given in the legends and also in *Material and Methods* section (paragraph *Statistical analyses and sample-sited estimation*) of the manuscript. The statistics section also specifies the software used (Prism).

Raw data is always presented in form of dot/bar plot combinations in the figures when n numbers are low (quantitative biochemical analyses).

As stated in the figure legends and also in the *Material and Methods* section (paragraph *Statistical analyses and sample-sited estimation*) of the manuscript, all data is mean plus/minus SEM except Figure 6C (% of total has no error) and Figure 9F (total error), as stated. Figure 5D shows both SD and SEM, as described in the legend.

The p-values for all findings are provided directly inside of the figures.

Note that, as also stated in the manuscript, most of our phenotypical analyses are of extremely high statistical significance (****) and that Prism 6 software does not provide numerical p-value data below 0.0001.

In all of these cases, solely **** thus is reported in the figures.

(For large datasets, or papers with a very large number of statistical tests, you may upload a single table file with tests, Ns, etc., with reference to sections in the manuscript.)

**Group allocation**

- Indicate how samples were allocated into experimental groups (in the case of clinical studies, please specify allocation to treatment method); if randomization was used, please also state if restricted randomization was applied
- Indicate if masking was used during group allocation, data collection and/or data analysis

Please outline where this information can be found within the submission (e.g., sections or figure legends), or explain why this information doesn’t apply to your submission:

No samples were allocated into any experimental groups.

**Additional data files (“source data”)**

- We encourage you to upload relevant additional data files, such as numerical data that are represented as a graph in a figure, or as a summary table
- Where provided, these should be in the most useful format, and they can be uploaded as “Source data” files linked to a main figure or table
- Include model definition files including the full list of parameters used
- Include code used for data analysis (e.g., R, MatLab)
- Avoid stating that data files are “available upon request”

Please indicate the figures or tables for which source data files have been provided:

A source data file (in Excel format) with numerical data covering the quantitative analyses shown in

Figure 1-Figure Supplement 2E-G

Figure 1-Figure Supplement 2E-G

Figure 1G-I

Figure 1N-P

Figure 3D-F

Figure 3G

Figure 5C,D

Figure 6B-D

Figure 7E

Figure 8D-F

Figure 8G

Figure 9D

Figure 9F

Figure 9I and

Figure 9J-L

is included.

All software used for analyses is reported in the Material and Method section of the manuscript.

IMARIS software parameters and settings for morphological analysis of neurons are also reported in the Material and Method section of the manuscript.
